# Challenges in developmental psychology, a focus on Sustainable Development

**DOI:** 10.3389/fpsyg.2022.1086458

**Published:** 2022-11-18

**Authors:** Peter Klaver, Katharina J. Rohlfing

**Affiliations:** ^1^Centre for Research and Development, University of Teacher Education in Special Needs, Zurich, Switzerland; ^2^Institute of Psychology, University of Zurich, Zurich, Switzerland; ^3^Faculty of Arts and Humanities, Paderborn University, Paderborn, Germany

**Keywords:** Open Science, Sustainable Development Goals (SDGs), Open Research Data, cultural context, developmental difference, methodological standards

## Introduction

Developmental psychology is traditionally of interdisciplinary nature with the aims to understand mechanisms of normative and individual changes and consistencies throughout the lifespan. The applied fields of developmental psychology target into health and educational sciences. The aims of developmental psychology cannot be reached without understanding and applying the underlying biological, senso-motoric, emotional, social, and cognitive processes that are intertwined with cultural context.

The field changes in dependence on internal and external factors. First, the increase in knowledge, in each of the associated areas can change the focus of interest in subfields or the whole field of developmental psychology. Second, methodological inventions can boost the development of certain subfields or the whole field of developmental psychology. Third, sudden changes in cultural context, by global events, such as strikingly demonstrated recently during the COVID-19 pandemic, affect the focus of interest in certain periods in life, or in interventions. Fourth, there is a solid claim for a bias in research on and from Western, Educated, Industrialized, Rich and Democratic (WEIRD) countries (Henrich et al., 2010), which affect inferences and impact from research in developmental psychology. Awareness toward the cultural context of research in developmental psychology, its limitations and generalizability, can change perspective and course of the field.

In the following Specialty Grand Challenge, we would like to sketch the most important challenges we observe within the field of developmental psychology, which are dependent on the above-mentioned areas of change: changes in knowledge and methodology, changes or bias in environment by global events and cultural context.

## Aims of the Specialty Grand Challenge

One of the key challenges we observe in developmental psychology relate to the Sustainable Development Goals (SDGs). The Agenda 2030 and the associated SDGs pose new challenges on science as a whole and as such on developmental psychology. The global goals to sustain the resources in the world's biosphere for future generations are balanced across the dimensions of social responsibility, ecological responsibility, and economic growth (United Nations Sustainable Development Group, 2019). Local or global actions at each of these dimensions can have an impact on the individual development and potentially on the normative development of people. Thus, they may affect individuals differently in childhood, youth, and adulthood. For example, youth in several countries have shown to take responsibility regarding SDGs to engage against climate change, against poverty, diversity, human rights, and democracy (Plan International UK, 2018), while Education for Sustainable Development is part of the curriculum in many countries. The development of youth may be affected alone by the responsibility they take, and the social changes they achieve may affect development of future generations. Participating more actively in a society and being encouraged by children's rights (UNICEF), children might require more support in developing skills that pertains to engagement in active citizenship from early on. The global context and youth's participation put novel requirements on youth's and children's care, caregivers as well as education.

Further, fast economic growth in urban areas as well as political conflicts can lead to migration waves toward these areas, which provides an impact on the health and education infrastructure in both rural and urban areas, which may in turn affect vulnerable populations disproportionally, and particularly children, elderly, and individuals with disabilities. Observations of the kind has been observed in several countries (Jampaklay et al., 2016; Holecki et al., 2020; Martín-Cano et al., 2020).

## Actions relating to SDGs

In our view, SDGs require actions in the field of developmental psychology regarding relevant methodologies and topics. We observe two windows of opportunity to take action on the impact of SDGs on developmental psychology. First, global developments in methodology of research, particularly Open Science and Open Research Data help to access, merge, and analyze data in understanding emotion, cognition, and social dynamics in development within cultural context. These approaches initiate new discoveries but require an understanding of the technological functions and their limitations. Second, digitalization has a global impact on the use of knowledge and skills in social, health, and educational systems, which induces challenges and boosts research in the field of developmental psychology.

Regarding the methodologies, we recommend for three levels of actions. First, research in developmental psychology needs to adopt to the principles of Open Science. The movement of Open Science has been seen as an important step toward managing resources at a global scale. Open Access of publications as implemented by Frontiers and other publishers, increases access to knowledge across the globe. However, sharing data by implementing FAIR (Findable, Accessible, Interoperable, and Reusable) principles on Open Research Data (ORD) would allow researchers across the globe to reuse data and connect own data to a larger pool of data than ever before. This could increase validation of evidence across different cultural contexts and could diversify and generalize theories on development. Simultaneously, methods can be further developed to adjust to novel contextual circumstances. Furthermore, a reuse of material, measures, procedures, and data contributes to more transparency and works against data loss, fosters collaborations, and generates reproducible workflows (Klein et al., 2018). With these methods, key issues relating to development and SDGs can be more easily studied, because contextual differences can be identified in larger datasets. Currently, Frontiers does not have a policy regarding ORD, but in our view, we could and should commit to such a policy within our research communities.

Second, research in developmental psychology should be committed to international standards regarding the adoption of research designs, conducting research, reporting and critical appraisal standards. The EQUATOR network currently provides 529 reporting guidelines for all types of research, which are relevant for social sciences and medicine, such as PRISMA (Page et al., 2021). Research communities, for example in neuroscience and medicine, have increased awareness toward such standards and thereby increased the quality of research (O'Brien et al., 2014; Song et al., 2017). It is important that research communities commit themselves toward such standards to increase reproducibility, transparency, and sustainability of research across the globe. In addition to reporting standards, the reviewing standards should be transparent and compliant with the guidelines. Several valuable critical appraisal tools have been developed, such as CASP or JBI checklists, which can support and standardize reviewing processes. By taking such standards more seriously, and with a higher commitment to them, globally, the rigor in addressing key questions relating to development can be pushed forward, and a sustainable knowledge base can be acquired.

Third, we observe strong innovations and methodological developments in areas, which now affect the field of developmental psychology. It will be important to increase awareness about applying technologies for specific solutions and specific contexts. A recent Research Topic on empirical research at a distance provides a good example of putting together novel and rapid methodical developments driven by COVID-19 pandemic but discussing their limitations thoroughly (Tsuji et al., 2022). In our view, it is important for novel methods to be presented in a comprehensive manner to provide informative access not only to procedures but also to its purposes and shortcomings. For this, Frontiers offers formats that might be used more often: Data Report, Technology and Code, Study Protocol, but also Brief Research Report could be used to introduce a technologically innovative method to describe novel possibilities of analysis. It should then involve a reflection about the used technology regarding ethics and privacy, its advantages, and disadvantages by addressing tradeoffs, for example between real world behaviors and in-lab controls. Examples of such technologies, involve techniques to record high resolution spatial and temporal data in more realistic settings such as Real-Time response measurements (Waldvogel and Metz, 2020), dyads in Experience Sampling Methods (Xia et al., 2022), Near Field Communication (Lorusso et al., 2018), Google Trends Research (Jun et al., 2018; Mavragani et al., 2018), and hyperscanning EEG (Kayhan et al., 2022). A debate on the use of these techniques may provide a window of opportunity for global research on development and SDGs.

Regarding topics, we observe a strong impact of digitalization on health and education systems. Health issues have become a global phenomenon. The way in which people at different stages during the life span have access to health care, receive information on health risks and preventions, and are at risk to health problems is not evenly distributed. Industrial pollution, access to nutrients, eating habits, digital literacy and consumption, access to information depend on societal, technological, and climate changes. These potential risk and resources can affect fetal, early development and parenting, as well as later periods in life. The increasing knowledge about the mechanisms and the more widespread possibilities to observe changes longitudinally in several areas around the world lead to a more detailed understanding of the development of health risks and the effect of interventions during life span. In our view, it is important that the knowledge about health risks at all ages and heterogeneous populations is well-documented and (digitally) accessible for use by professional health practitioners.

Along similar lines, we observe that education undergoes changes depending on technological development, particularly digital information technology. Access to information and knowledge about education governance, access to education during political, societal and climate change can all affect the way in which children develop and the way in which communities and families are able to support the development of children. The current access to larger datasets has the potential to facilitate research about the psychological development on these topics. We therefore encourage the research on human development, health, and education specifically concerned with the influence of social and technological change in dynamic cultural contexts.

## Discussion

The main challenges that we observe are related to the global movements in science and society, which affect the course that developmental psychology takes. In the past, developmental psychology has been dominated by European and North American scientists, followed by important research from Japan (Norimatsu, 2018). Now, researchers from an increasing number of countries across the world, but particularly China deliver important insights into the field of developmental psychology. Further, the Agenda 2030, the global issues relating to health and education, the Open Science movement, the increasing knowledge about developmental psychology from other counties, the ability to merge greater datasets provide the possibility to understand development at a different level. Our view is indeed in line with these developments, first because we see a chance that normative and individual changes in development can be better understood at both a global and local level. Second, because we trust that applied scientists can address that knowledge. Their task is to improve, for example, health care, health risk prevention and education in cooperation with professionals and providers of social support.

Nevertheless, we are aware of the subjective nature of our view. The foundation of it was mainly based on the set of Research Topics that we evaluate at the Frontiers in Psychology Specialty Section Developmental Psychology, the manuscript we receive in our role as Section Chief Editors, as well as our role we have as professionals at our universities and the research communities we work in. We are also aware of the diversity of ethical and cultural values, which drives our vision of research on developmental psychology and the visions of all researchers in the field. The hope that shared values on the necessity to continue research on developmental psychology in the light of Sustainable Development remains.

## Author contributions

All authors listed have made a substantial, direct, and intellectual contribution to the work and approved it for publication.

## Conflict of interest

The authors declare that the research was conducted in the absence of any commercial or financial relationships that could be construed as a potential conflict of interest.

## Publisher's note

All claims expressed in this article are solely those of the authors and do not necessarily represent those of their affiliated organizations, or those of the publisher, the editors and the reviewers. Any product that may be evaluated in this article, or claim that may be made by its manufacturer, is not guaranteed or endorsed by the publisher.
